# The battle on time, money and precision: Da[^18^F] id vs. [^68^Ga]liath

**DOI:** 10.1007/s00259-020-04961-1

**Published:** 2020-07-26

**Authors:** Sabri Sahnoun, Patrick Conen, Felix M. Mottaghy

**Affiliations:** 1 Department of Nuclear Medicine, University Hospital RWTH Aachen University, Pauwelsstr. 31, 52074 Aachen, Germany; 2 Center of Integrated Oncology (CIO), Universities of Aachen, Bonn, Cologne, and Duesseldorf, Cologne, Germany; 3 Department of Radiology and Nuclear Medicine, Maastricht University Medical Center (MUMC+), P. Debeylaan 25, 6229 HX Maastricht, P.O. Box 5800, 6202 AZ Maastricht, Netherlands

Since its first introduction some 20 years ago, [^68^Ga]-radiolabelled somatostatin receptor agonists (SSAs) have gained high importance in the management of neuroendocrine tumours [1], especially due to the better delineation of small structures compared with the licensed product [^111^In]In-pentetreotide ([^111^In]In-DTPA-[D-Phe^1^]-octreotide, OctreoScan**®**) [2, 3]. In clinical routine PET imaging of the somatostatin receptor (SSTR) in patients with neuroendocrine tumours (NETs) employing on [^68^Ga]Ga-DOTA-peptides such as [^68^Ga]Ga-DOTATOC or [^68^Ga]Ga-DOTATATE is the gold standard [4]. Current guidelines recommend a preplacement of [^111^In]In-DTPA-octreotide by [^68^Ga]-labelled SSAs [5]. The theranostic pair with [^177^Lu]Lu-DOTATOC or [^177^Lu]Lu-DOTATATE as radiotracers for peptide receptor radionuclide therapy (PRRT) has been established [6].

The big advantage of the positron-emitting radiometal [^68^Ga] is its availability from a [^68^Ge]/[^68^Ga] generator, providing an independence from a local cyclotron installation. This aspect and the fact that some of the available generators have received regulatory approval have spread [^68^Ge]/[^68^Ga] generators and their production facilities worldwide in recent years. Nevertheless [^68^Ga] has at the same time several disadvantages. With a relatively short half-life (68 min) [7], the possibilities for decentralized production are very limited and restrict its use in centres that have no adequate radiopharmacy. Another limiting point is that the low overall activity yield per synthesized batch is only sufficient for a maximum of four patients per production. In addition to logistical disadvantages, [^68^Ga] also has drawbacks based on its physical properties. [^68^Ga] is a long-range positron emitter (> 1 mm) with a relatively high positron energy (*E*_mean_ = 0.83 MeV) which corresponds to a relatively long positron range (*R*_mean_ = 3.5 mm) resulting in relatively blurred images due to a suboptimal spatial resolution compared with the radiohalogen [^18^F] [8, 9]. For these reasons, the implementation of especially [^18^F] labelling of SSAs for PET imaging of NETs has been studied.

Concerning clinical PET imaging, [^18^F] is the most commonly used positron-emitting radiohalogen. Unlike [^68^Ga], it offers several logistic and physical benefits. [^18^F] with a half-life of 109.77 min [10] can be produced in large amounts by cyclotrons, and locally synthesized PET tracers can consecutively be easily transported over a longer distance to hospitals and departments without cyclotron (satellite concept). Another advantage is that [^18^F] is a short-range positron emitter (< 1 mm) with a low positron energy (*E*_mean_ = 0.25 MeV) and a corresponding shorter positron range (*R*_mean_ = 0.6 mm) resulting in a higher spatial resolution [8, 9].

To meet the high radiotracer demand in PET imaging of NETs, the group of Hans-Jürgen Wester evaluated already more than 10 years ago a fluorine-18-labelled somatostatin receptor agonist, Gluc-Lys([^18^F]FP)-TOCA, which showed a superior diagnostic performance compared with [^111^In]In-DTPA-octreotide. Due to its complex multistep synthesis and the poor radiochemical yield, this tracer was not implemented in clinical practice [11–13]. The chemical advantages in using silicon-fluoride acceptor (SiFA) chemistry allowing a simple and mild radiolabeling procedure without generating radiochemical by-products or derivatives were demonstrated [14]. A promising fluorine-18-based SSA tracer, [^18^F]SiFAl*in*-TATE [15], was explored in a patient with metastatic NET and compared with [^68^Ga]Ga-DOTATOC. This case report demonstrated that the uptake of [^18^F]SiFAl*in*-TATE in healthy and tumour tissue is in the same range as [^68^Ga]Ga-DOTATOC [16]. A tracer synthesizing method which combines the advantages of a chelator-based radiolabelling and the properties of [^18^F] was developed in 2009 [17]. Laverman et al. introduced [^18^F]AIF-NOTA-octreotide and compared it with [^111^In]In-DTPA-octreotide and [^68^Ga]Ga-NOTA-octreotide in preclinical models. This comparison confirmed a high in vitro binding affinity of [^18^F]AIF-NOTA-octreotide towards SSTR2 [18, 19]. In 2019, the first clinical experience with [^18^F]AIF-NOTA-octreotide in 22 NET patients was reported showing that the tracer displays a favourable biodistribution and provides an excellent detection of tumoural lesions with a high tumour-to-background ratio; however, there was no comparison with a [^68^Ga]-labelled SSA [20]. It took almost 10 years until an automated GMP compliant production of [^18^F]AIF-NOTA-octreotide was published and a GMP grade precursor became commercially available [21].

In this issue, a systematic biodistribution study of [^18^F]AIF-NOTA-octreotide as well as a first comparison to the clinical standard [^68^Ga]Ga-DOTATATE is presented by the group from Leuven, Belgium, using this automated GMP compliant production (Pauwels et al.: [^18^F]AlF-NOTA-octreotide PET imaging: biodistribution, dosimetry and first comparison with [^68^Ga]Ga-DOTATATE in neuroendocrine tumour patients, in print). While the acquisition of [^68^Ga]Ga-DOTATATE is recommended to be started about 45–60 min p.i. [22, 23], the authors showed that 120 min p.i. reveal the best target-to-background ratio for [^18^F]AIF-NOTA-octreotide imaging. This aspect has to be taken into account in the logistic planning of the scanning if the tracer would be introduced into routine. The very small group of patients does not allow the conclusion that [^18^F]AIF-NOTA-octreotide is superior to [^68^Ga]Ga-DOTATATE, but it can be stated that it is certainly not inferior to the gold standard. The favourable dosimetry, biodistribution, kinetics and binding affinity/tumour targeting hold promise for a competitive compound in the management of NET patients.

In conclusion we think that the easy to synthesize and GMP compliant tracer [^18^F]AIF-NOTA-octreotide has definitely the potential to become the rising star in SSR imaging and might prove to also have economical advantages.
